# Long-term left ventricular thrombosis resolution in patients receiving vitamin k antagonists: a multicenter observational study

**DOI:** 10.1007/s11739-025-03922-6

**Published:** 2025-04-03

**Authors:** Emanuele Valeriani, Giulia Astorri, Arianna Pannunzio, Daniele Pastori, Ilaria Maria Palumbo, Danilo Menichelli, Marco Paolo Donadini, Davide Santagata, Katarzyna Satula, Erica De Candia, Luca D’Innocenzo, Antonella Tufano, Rossella Marcucci, Martina Berteotti, Antonio Chistolini, Francesco Dragoni, Tommaso Bucci, Walter Ageno, Cecilia Becattini, Pasquale Pignatelli

**Affiliations:** 1 Department of General Surgery and Surgical Specialty Paride Stefanini, Sapienza University of Rome, Rome, Italy; 2 Department of Infectious Disease, Azienda Ospedaliero-Universitaria Policlinico Umberto I, Viale del Policlinico, 155, Rome, Italy; 3 Department of Clinical Internal, Anesthesiological and Cardiovascular Sciences, Sapienza University of Rome, Rome, Italy; 4 Department of Medicine and Surgery, University of Insubria, Varese, Italy; 5 Internal Vascular and Emergency Medicine - Stroke Unit, University of Perugia, Perugia, Italy; 6 Department of Diagnostic Imaging, Radiotherapy, Oncology and Haematology, Hemorrhagic and Thrombotic Diseases Center, Fondazione Policlinico Universitario A. Gemelli IRCCS, Rome, Italy; 7 Department of Clinical Medicine and Surgery, Federico II University of Naples, Naples, Italy; 8 Department of Clinical and Experimental Medicine, University of Florence and Careggi Hospital, Florence, Italy; 9 Hematology, Department of Translational and Precision Medicine, Sapienza University of Rome, Rome, Italy

**Keywords:** Acenocoumarol, Anticoagulants, Heparin, Venous thromboembolism, Warfarin

## Abstract

Optimal duration of anticoagulant therapy for left ventricular thrombous (LVT) is unclear. The aim of this study is to evaluate effectiveness and safety of vitamin K antagonists (VKAs) up to 12 months in patients with LVT. Patients diagnosed with LVT between 2011 and 2023 and treated with VKAs until LVT resolution or up to 12 months were enrolled in a retrospective cohort study. Primary outcome included on-treatment LVT resolution, secondary outcomes acute ischemic stroke, myocardial infarction, peripheral embolism, and major and clinically relevant non-major bleedings during the 12-month follow-up. Ninety patients were included. Median age was 66 years and 78.9% were male. Mean time in therapeutic range was 61% and 32.9% of patients received VKA monotherapy, with the remaining concomitant antiplatelet treatment. The 3, 6, 12 months cumulative incidences of LVT resolution were 27% (95% confidence intervals -95%CI-, 18%–36%), 47% (95%CI 36%–57%), and 70% (95% CI 60%–79%), respectively. At Cox regression model, reduced left ventricular ejection fraction (Hazard Ratio 0.48; 95%CI 0.24–0.95) and left-ventricular aneurysms (Hazard Ratio 0.44; 95%CI 0.22–0.88) were associated with reduced LVT resolution. One patient developed an acute ischemic stroke and one an acute myocardial infarction. Two patients developed a major and four a clinically relevant non-major bleeding. Incidence of LVT resolution appeared to be higher at 12 than at 3 and 6 months of follow-up, and the rates of on-treatment acute arterial and bleeding events were low. Reduced left ventricular ejection fraction and left-ventricular aneurysm appeared to be associated with a lower rates of LVT resolution.

**Supplementary Information:**

The online version contains supplementary material available at 10.1007/s11739-025-03922-6.

## Background

Left ventricular thrombus (LVT) is a not negligible complication of acute or chronic cardiomyopathy (e.g., acute coronary syndrome) and represents a life-threatening condition with a high risk of stroke and systemic embolism [1]. These risks appeared to be higher in patients with protruding and mobile thrombus, in patients with LVT persistence and/or recurrence, and in patients not receiving anticoagulation [2–7]. Thrombus recurrence after resolution may also occur in 10% to 20% of patients being associated with a high embolic risk [8–10].

An accurate clinical and echocardiographic evaluation as well as a proper anticoagulant therapy is mandatory to ameliorate patients’ prognosis. International guidelines recommend oral anticoagulant therapy with vitamin K antagonists (VKAs) for at least three to six months with weak grade of evidence, but the optimal antithrombotic regimen to achieve thrombus resolution and to prevent cardioembolic events has not been established [1, 11, 12]. Indeed, available data on therapeutic management of LVT are sparse and mostly derived from small observational studies with highly variable baseline patients’ characteristics, type and duration of treatment, and rates of clinically relevant outcomes during follow-up. Anticoagulant therapy with VKAs seemed to afford for a high rate of thrombus resolution (from less than 50% up to more than 80% of patients) and for a low rate of stroke and systemic embolisms (less than 10% of treated patients) with an acceptably low risk of bleeding events [11, 13, 14]. The optimal treatment duration is a further matter of debate with some authors suggesting the possible benefits of extending anticoagulation beyond the first three to six months in specific patients at high thrombotic risk [15–18]. Whether specific patients’ characteristics may be associated with thrombus persistence and may justify a longer course of anticoagulant therapy have to be yet evaluated.

Therefore, the objectives of our study were to evaluate the effectiveness and safety of a course of VKAs therapy until thrombus resolution or up to 12 months in patients with LVT and to identify patients’ characteristics associated with thrombus resolution.

## Methods

This study is reported in accordance with the Strengthening the Reporting of Observational Studies in Epidemiology statement for observational studies [19].

### Study and patients’ characteristics

In this retrospective study, patients referred to 6 Italian Thrombosis Centers from 2011 through 2023 for LVT development were included in the analysis if they were treated with VKAs until thrombus resolution or for up to 12 months, and if data on thrombus resolution during follow-up were available. Exclusion criteria included anticoagulant therapy different from VKAs (e.g., direct oral anticoagulants – DOACs), treatment duration < 3 months, other indication for VKAs than LVT (e.g., venous thromboembolism, mechanical prosthetic valve, right ventricle, or atrial thrombosis), and unavailability of relevant data. All included patients consented the use of their data for study purposes.

The diagnosis of LVT was accepted if confirmed by contrast-enhanced transthoracic echocardiography, computed tomography, or magnetic resonance imaging. INR values were regularly monitored, and therapeutic dosage were managed to maintain patients’ specific INR values as recommended by the attending physician.

The following data were collected in an electronic dataset: demographic and cardiovascular risk factors (e.g., age, sex category, history of hypertension, diabetes mellitus, dyslipidemia), risk factors for LVT development (e.g., ischemic, dilated, and hypertrophic cardiomyopathy as defined by clinical and echocardiographic characteristics), echocardiographic characteristics (e.g., left-ventricular ejection fraction – LVEF –, left-ventricular aneurysm, valves alterations), type of VKAs (i.e., warfarin, acenocoumarol), duration of treatment, time in therapeutic range (TTR – Rosendaal method) [20], concomitant antiplatelet therapy, and outcomes of interest.

### Outcomes

The primary outcome included on-treatment thrombus resolution during the 12 months of follow-up. Thrombus resolution was defined by LVT disappearance at imaging (transthoracic echocardiography, computed tomography, or magnetic resonance imaging) performed as recommended by the attending physician. Secondary outcomes included objectively documented acute ischemic stroke, acute myocardial infarction, acute peripheral embolism, and major and clinically relevant non-major bleedings as defined by the International Society of Thrombosis and Haemostasis (ISTH) criteria during the 12 months of follow-up [21, 22].

### Statistical analysis

Baseline characteristics of the enrolled population are reported as descriptive statistics. Continuous variables are expressed as mean (standard deviation) or median (interquartile range – IQR –), according to data distribution after applying the Shapiro–Wilk test. Categorical variables are expressed as counts and percentages. Patients were sorted in two groups based on the presence of thrombus resolution during follow-up or not. Continuous variables were compared using the Student's t test or the Mann–Whitney U test, and categorical variables were compared using the chi-squared or Fisher's exact tests, as appropriate. The frequency of thrombus resolution was expressed as 3-, 6-, and 12-month cumulative incidence with 95% confidence intervals (95%CIs), while the frequency of other outcomes was descriptively reported. Kaplan–Meier curves were calculated for thrombus resolution in patients with a preserved (i.e., ≥ 50%), mildly reduced (i.e., 40–49%), and reduced (i.e., < 40%) LVEF and in patients with or without left-ventricular aneurysm. Cox proportional hazards model was used to identify potential predictors for thrombus resolution at 12 months of follow-up. We decided to develop the model selecting a priori the variables of interest to be included as predictors, based on available data and expert clinical reasoning [1, 9, 10, 23–25]. The following variables were chosen a priori as considered clinically relevant for the outcome of interest and included in the model: age, male sex, LVEF, presence of left-ventricular aneurysms, and TTR < 50% [1, 9, 10, 25]. These variables were included in the model if data were available in at least 75% of the overall population and no collinearity were identified. RStudio (version 2023.09.1 + 494, R Core Development Team, Vienna, Austria) was used for the analysis [26].

## Results

### Baseline patient characteristics

Of 112 patients evaluated for inclusion, 22 were excluded as they received VKAs treatment for a duration shorter than 3 months (3 patients) or because relevant information was missing (19 patients). Finally, 90 patients were included in the primary analysis.

Table 1 reports the baseline characteristics of the overall population and according to LVT resolution, which was documented in 65 patients (72.2%). Overall, the mean age was 66 years (± 13 years), and 71 (78.9%) patients were male without significant differences about age and sex category between patients with and without thrombus resolution (Table 1). Furthermore, no difference was observed in common cardiovascular risk factors and in the echocardiographic characteristics between the two groups except for the presence of left-ventricular aneurysm that was more frequent in patients who did not achieve LVT resolution (44.0% versus 16.4%) (Table 1).

**Table 1:** Baseline patients’ characteristics

Variables	Overall *n* = 90	LVT persistence *n* = 25	LVT resolution *n* = 65	*p*-values
Mean age, years (SD)	66 (13)	63 (14)	67 (12)	0.195
Male sex, *n* (%)	71 (78.9)	22 (88.0)	49 (75.4)	0.305
*Cardiovascular risk factors and comorbidities*				
Smoking history, *n* (%)^*^	33 (42.9)	13 (61.9)	20 (35.7)	0.070
Arterial hypertension, *n* (%)	55 (61.1)	15 (60.0)	40 (61.5)	1.000
Diabetes mellitus, *n* (%)	26 (28.9)	5 (20.0)	21 (32.3)	0.371
Dyslipidemia, *n* (%)^*^	52 (58.4)	14 (56.0)	38 (59.4)	0.959
Atrial fibrillation, *n* (%)^*^	18 (20.2)	8 (32.0)	10 (15.6)	0.151
Cerebrovascular disease, *n* (%)^*^	12 (13.5)	4 (16.0)	8 (12.5)	0.929
Peripheral arterial disease, *n* (%)^*^	5 (5.7)	0 (0.0)	5 (7.9)	0.347
*Clinical and echocardiographic characteristics*				
Coronary artery disease, *n *(%)	73 (81.1)	20 (80.0)	53 (81.5)	1.000
Dilated cardiomyopathy, *n* (%)	29 (32.2)	12 (48.0)	17 (26.2)	0.083
Hypertrophic cardiomyopathy, *n* (%)	10 (11.1)	2 (8.0)	8 (12.3)	0.835
Mitral insufficiency, *n* (%)^*^	50 (61.0)	16 (69.6)	34 (57.6)	0.457
Mitral stenosis, *n* (%)^*^	3 (3.7)	0 (0.0)	3 (5.1)	0.655
Aortic insufficiency, *n* (%)^*^	16 (19.5)	3 (13.0)	13 (22.0)	0.540
Aortic stenosis, *n* (%)^*^	1 (1.2)	0 (0.0)	1 (1.7)	1.000
LVEF, *n* (%)^*^				0.124
> 50%	16 (19.0)	2 (8.7)	14 (23.0)	
40%–49%	31 (36.9)	7 (30.4)	24 (39.3)	
< 40%	37 (44.0)	14 (60.9)	23 (37.7)	
Left-ventricular aneurysm, *n* (%)^*^	21 (24.1)	11 (44.0)	10 (16.1)	0.013
Site of LVT, *n* (%)^*^				1.000
Apical	85 (97.7)	23 (95.8)	62 (98.4)	
Other	2 (2.3)	1 (4.2)	1 (1.6)	
Diagnostic method				0.989
Trans-thoracic echocardiography	55 (61.1)	16 (64.0)	39 (60.0)	
Magnetic resonance imaging	8 (8.9)	2 (8.0)	6 (9.2)	
Computed tomography	4 (4.4)	1 (4.0)	3 (4.6)	
Not reported	23 (25.6)	6 (24.0)	17 (26.2)	
*Lab values*				
Median Hemoglobin, g/dl [IQR]^°^	14.2 [13.0, 15.7]	13.8 [12.9, 15.9]	14.2 [13.3, 15.4]	0.975
Median platelets, *10^3^/ul [IQR]^°^	216 [185, 273]	191 [156, 225]	228 [195, 282]	0.049
Median creatinine, mg/dl [IQR]^°^	1.10 [0.95, 1.40]	1.01 [0.99, 1.14]	1.10 [0.90, 1.40]	0.751
Median AST, IU/l [IQR]^$^	25 [21, 49]	21 [17, 48]	27 [24, 48]	0.463
Median ALT, IU/l [IQR]^$^	30 [18, 45]	44 [23, 58]	29 [19, 45]	0.680
Mean 12-month TTR, [IQR]^$^	61 (17)	62 (17)	61 (18)	0.803
*Therapy*				
Concomitant aspirin, *n* (%)^*^	50 (61.0)	15 (68.2)	35 (58.3)	0.579
Concomitant p2y12, *n* (%)^*^	41 (50.6)	14 (66.7)	27 (45.0)	0.145
DAPT, *n* (%)^*^	34 (44.2)	13 (61.9)	21 (37.5)	0.096
VKA monotherapy, *n* (%)^*^	27 (32.9)	5 (22.7)	22 (36.7)	0.355
*12-month outcomes*				
Acute ischemic stroke, *n* (%)	1 (1.1)	0 (0.0)	1 (1.5)	1.000
Acute myocardial infarction, *n* (%)	1 (1.1)	1 (4.0)	0 (0.0)	0.636
Acute limb ischemia, *n* (%)	0 (0.0)	0 (0.0)	0 (0.0)	NA
Major bleeding, *n* (%)	2 (2.3)	0 (0.0)	2 (3.3)	0.898
CRNMB, *n* (%)	4 (4.4)	1 (4.0)	3 (4.6)	1.000

*CRNMB* clinically relevant non-major bleeding; *LVEF* left ventricular ejection fraction; *LVT* left ventricular thrombus; *TTR* time in therapeutic range; *VKAs* vitamin K antagonists

^*^Data available for > 75% of patients

^°^Data available for 50%–75% of patients

^$^Data available for < 50% of patients

Overall, treatment duration was ≤ 3 months in 10.0%, between 3 and 6 months in 7.8% and between 6 and 12 months in 82.2% of patients with a median treatment duration of 12 months (IQR, 8 to 12 months). TTR values were available for 50.0% of patients. The overall quality of VKAs therapy was low with a mean TTR of 61% (warfarin, 42 patients; acenocoumarol, 3 patients). Mean TTR values were similar in patients with and without LVT resolution during follow-up (62% versus 61%).

Similar proportions of patients with or without LVT resolution received acetylsalicylic acid (68.2% versus 58.3%), P2Y12 inhibitors (66.7% versus 45.0%), dual antiplatelet therapy (61.9% versus 37.5%), or VKAs monotherapy (22.7% versus 36.7%).

### Cumulative incidence of thrombus resolution and Cox proportional hazard model

During follow-up, 27% of patients (95%CI, 18% to 36%) had a LVT resolution at 3 months, 47% of patients (95%CI, 36% to 57%) at 6 months, and 70% of patients (95%CI, 60% to 79%) at 12 months of VKA therapy.

At multivariable Cox proportional hazard analysis (Table 2), reduced LVEF (Hazard Ratio, 0.48; 95%CI, 0.24 to 0.95) and left-ventricle aneurysm (Hazard Ratio, 0.44; 95%CI, 0.22 to 0.88) appeared to be associated with a reduced hazard of thrombus resolution.

**Table 2:** Univariable and multivariable COX regression models at 12 months of follow-up

Variables	Hazard ratio (95% CI)	Hazard ratio (95% CI)
Age	1.00 (0.99–1.00)	1.02 (0.99–1.04)
Male sex	0.70 (0.39–1.20)	0.74 (0.41–1.34)
Moderately reduced LVEF Reduced LVEF	0.64 (0.33–1.25) **0.44 (0.22**–**0.85)**	0.73 (1.37–1.42) **0.48 (0.24**–**0.95)**
Left-ventricle aneurysm	**0.42 (0.21**–**0.84)**	**0.44 (0.22**–**0.88)**

Statistically significant results were reported in bold

Data for all these variables was available for 84 patients. Variance inflation factor values: age, 1.04; male sex, 1.05; moderately reduced LVEF, 1.69; reduced LVEF, 1.71; left-ventricle aneurysm, 1.01

**Fig. 1:**
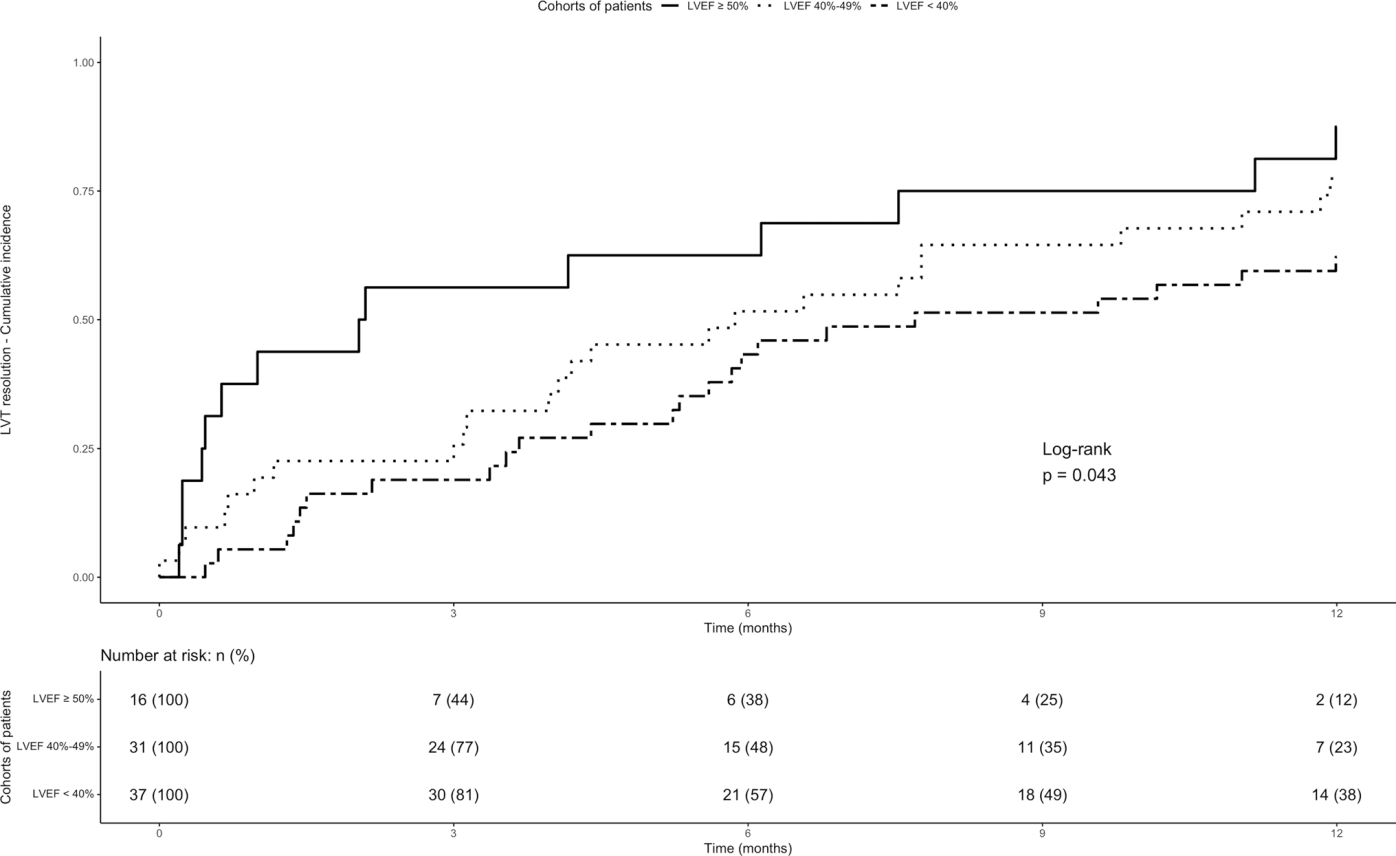
Incidence of LVT resolution in patients with different left-ventricle ejection fraction. Data were available for 84 patients. *LVEF* left ventricular ejection fraction; *LVT* left ventricular thrombosis

**Fig. 2:**
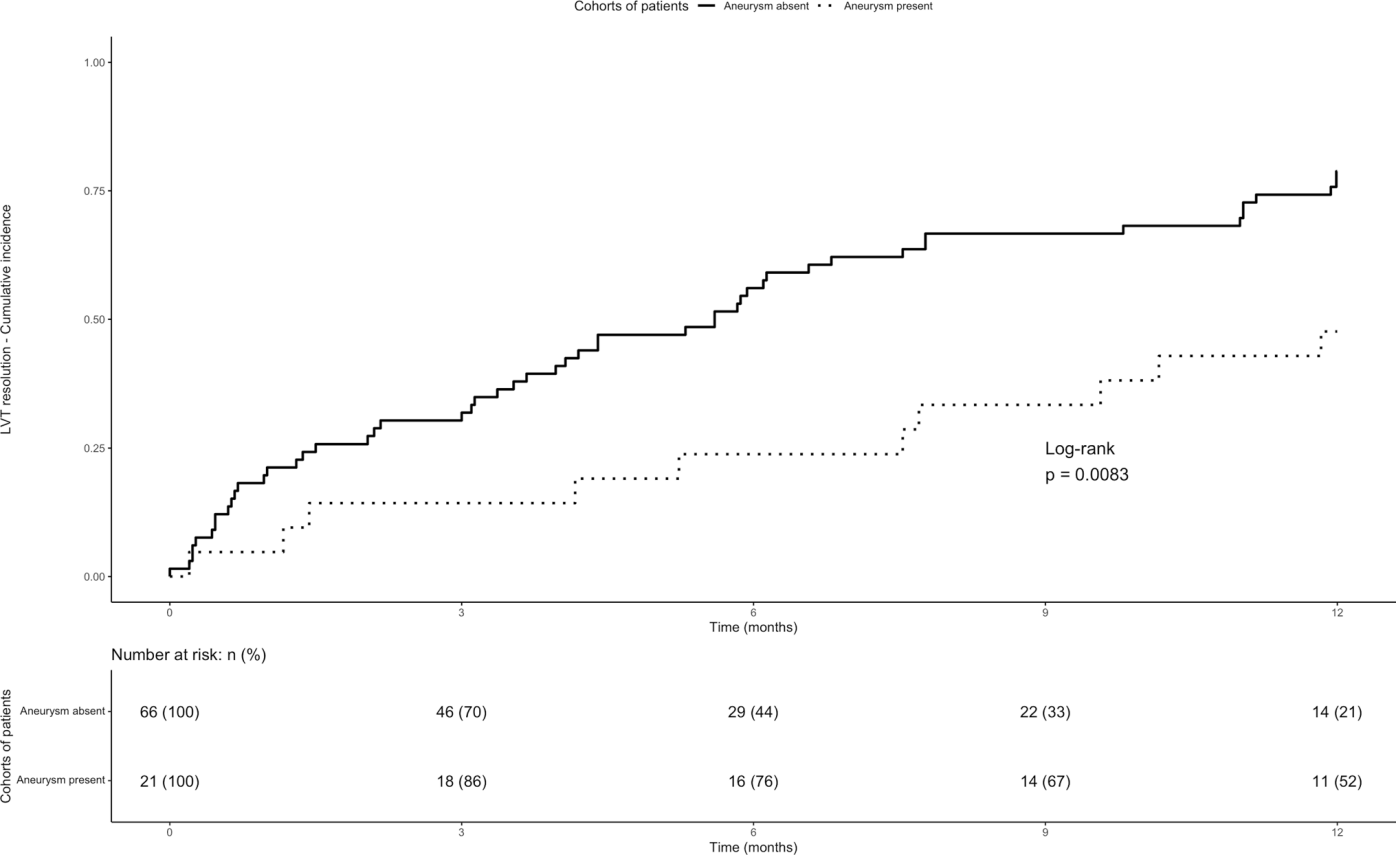
Incidence of LVT resolution in patients with or without left-ventricle aneurysm. Data were available for 87 patients. *LVT* left ventricular thrombosis

A graphic representation of cumulative incidences of LVT resolution by LVEF and by the presence of left-ventricular aneurysm or not is shown in Figs. 1 and 2.

### Arterial and bleedings events

Overall, one patient who achieved LVT recanalization developed an acute ischemic stroke one month after treatment discontinuation and one patient developed an on-treatment acute myocardial infarction after 7 months of VKAs therapy.

Two patients developed an on-treatment major bleeding (one cerebral bleeding after 9 months and one lower gastrointestinal bleeding after one month of VKAs therapy) and four patients developed an on-treatment clinically relevant non-major bleeding (one genitourinary bleeding after 5 months, one oral mucosa bleeding after 3 months, and two epistaxis after 3 and 4 months of VKAs therapy).

## Discussion

Our study provides an overview of current management and outcomes of patients with LVT. Less than one-third of patients did not achieve LVT resolution at 12 months with higher incidence of LVT resolution at 12 than at 3 and 6 months of follow-up. The presence of reduced LVEF and of left-ventricular aneurysm was associated with a lower incidence and hazard of LVT resolution. A low proportion of patients developed systemic embolism and the proportion of patients with on-treatment major and clinically relevant bleedings beyond the first three months of treatment was acceptably low.

In recent years, several observational studies and a few randomized controlled trials reported data on the efficacy and safety of anticoagulant therapy for the treatment of LVT (from less than 10 to about 250 anticoagulated patients) [13, 14]. The administration of therapeutic doses of intravenous heparin for 1 to 3 weeks appeared to provoke acceptable rate of LVT resolution [27, 28]. VKA therapy up to 6 months also proved its efficacy in terms of both LVT resolution (up to 80%) and prevention of acute arterial embolism (less than 10%) [13, 14]. Despite no studies compared different treatment durations, longer than recommended course of therapy was administered in some cases suggesting the possible benefits of extended anticoagulation [17, 18].

The relatively low incidence of LVT resolution observed in our study may be due to the high prevalence of mildly reduced (36.9%) and reduced (44.0%) LVEF and to the quite low quality of anticoagulation (61%), as a significant reduction of ischemic events is usually observed for TTR > 70% [29]. It should be acknowledged, however, that the median TTR found in our study was similar with the one reported in recent randomized trials on atrial fibrillation and venous thromboembolism (from 55 to 65%) [30]. Furthermore, our study confirms and extends previous data showing that the incidence of thrombus resolution is higher at 12 than at 3 months and 6 months of follow-up, with a prevention of arterial events and an acceptably low rate of bleeding events [17, 18]. Whether specific subgroups of patients with LVT are at higher thrombotic risk and may benefit from longer course of anticoagulant therapy remains unclear [1]. Indeed, the identification of patient characteristics associated with LVT resolution may further guide therapeutic decision. Even if data after treatment discontinuation are not available, our results suggest that a longer course of anticoagulant therapy may be justified in patients at higher thrombotic risk as those with a reduced LVEF and in patients with left-ventricular aneurysm.

Few data from randomized trial and observational studies are available for DOACs in this setting [1, 31]. While awaiting the results of ongoing studies, the use of DOACs should be actually considered off-label and possibly used with caution in specific subgroups of patients (e.g., low quality of treatment with VKA or impractical INR monitoring) [1].

Our study has some limitations that warrant discussion. First, the number of included patients was relatively low not being completely representative of this patient population nor allowing to perform sensitivity analysis in specific subgroup of patients (e.g., different causes of LVT, different classes of LVEF). We acknowledge the possibility of selection bias as some patients were excluded due to the lack of relevant data and as the use of anticoagulant treatment different than VKAs was an exclusion criterion. However, the current cohort of patients with LVT is derived from 6 Italian Thrombosis Centers where they were regularly monitored and where clinical outcomes were regularly collected during follow-up. Second, the retrospective nature of the study did not allow the collection of additional patient characteristics associated with LVT resolution. Third, diagnostic and treatment decisions were entirely at the discretion of attending clinicians and no diagnostic or therapeutic algorithms were provided hampering to draw firm conclusions on the effectiveness and safety of different anticoagulant strategies. In this regard, the long study period introduces potential variability in clinical practices, treatments, and technologies over time affecting the consistency of care and diagnostic methods. The difference in these latter may have affected outcome assessment due to their variable diagnostic power. It should be also acknowledged that the LVT resolution time was approximated using data from the available radiological imaging performed during follow-up. Furthermore, baseline characteristics and concomitant medications may have affected decisions about anticoagulation. Finally, the low number of events and the lack of specific characteristics in few patients did not allow the evaluation of further potential confounders for LVT resolution as well as arterial and bleeding events could be just descriptively reported.

## Conclusions

The incidence of LVT resolution appeared to be higher at 12 months than at 3 and 6 months of follow-up with a low proportion of on-treatment acute arterial and bleeding events. Reduced LVEF and left-ventricular aneurysm appeared to be associated with a lower incidence and hazard of LVT resolution. Nevertheless, the optimal management of patients with LVT is still to be found.

## Supplementary Information

Below is the link to the electronic supplementary material.

Supplementary file1 (DOCX 16 KB)

## Data Availability

All data generated or analysed during this study are included in this published article [and its supplementary information files].
