# Supplementary material for: Long-term left ventricular thrombosis resolution in patients receiving vitamin k antagonists: a multicenter observational study
Source: Intern Emerg Med. 2025 Apr 3;20(4):1069–76. doi: 10.1007/s11739-025-03922-6 (PMC12130138; doi:10.1007/s11739-025-03922-6)
Supplement: Supplementary file 1 — Supplementary file1 (DOCX 16 KB) [file 11739_2025_3922_MOESM1_ESM.docx]

**Supplementary Table 1. Shapiro-Wilk normality test for evaluated variables**

| **Variables** | **W** | **p-values** |
| --- | --- | --- |
| Age | 0.98016 | 0.1853 |
| Hemoglobin values | 0.95399 | 0.04306 |
| Platelet count | 0.86124 | 4.383e-05 |
| Creatinine values | 0.733 | 2.191e-08 |
| AST values | 0.4857 | 2.614e-09 |
| ALT values | 0.75283 | 1.668e-06 |
| TTR values | 0.98151 | 0.681 |
